# Dihydromyricetin Protects Intestinal Barrier Integrity by Promoting IL-22 Expression in ILC3s through the AMPK/SIRT3/STAT3 Signaling Pathway

**DOI:** 10.3390/nu15020355

**Published:** 2023-01-10

**Authors:** Jie Zhou, Jing Yue, Yu Yao, Pengfei Hou, Ting Zhang, Qianyong Zhang, Long Yi, Mantian Mi

**Affiliations:** Research Center for Nutrition and Food Safety, Chongqing Key Laboratory of Nutrition and Food Safety, Chongqing Medical Nutrition Research Center, Institute of Military Preventive Medicine, Third Military Medical University, Chongqing 400038, China

**Keywords:** dihydromyricetin, intestinal barrier, IL-22, SIRT3

## Abstract

Background: Previous studies indicate that dihydromyricetin (DHM) could alleviate intestinal inflammation and improve intestinal barrier integrity, yet the underlying mechanism remains obscure. Methods: *C57BL/6* male mice were fed with a control diet, high-fat diet (HFD), or HFD + DHM diet for 12 weeks. The intestinal permeability and expression of intestinal tight junction (TJ) protein were detected to evaluate the effects of DHM on intestinal barrier integrity. The interleukin 22 (IL-22) production of group 3 innate lymphoid cells (ILC3s) in small intestine lamina propria was tested to clarify the effects of DHM on ILC3s. In addition, an MNK3 cell line, which expresses the same transcription factors and cytokines as ILC3, was used to investigate the molecular mechanism under DHM-induced IL-22 expression. Results: DHM effectively protected HFD-fed mice against intestinal barrier destruction by promoting ILC3 activation and IL-22 secretion, and IL-22 expression increased the expression levels of TJ molecules to protect intestinal barrier integrity. Moreover, DHM increased activation of the AMPK/SIRT3/STAT3 pathway, which in turn promoted IL-22 expression in MNK3 cells. Conclusions: DHM improved IL-22 production in ILC3 cells to alleviate HFD-induced intestinal barrier destruction via the AMPK/SIRT3/STAT3 pathway.

## 1. Introduction

The intestinal barrier, the largest boundary between the human body and the environment, controls the absorption of essential dietary nutrients and resistance to ingested toxins and microbes. A high-fat diet (HFD) has been found to exacerbate intestinal barrier dysfunction and enhance the risk of developing intestinal inflammation [1]. Interleukin-22 (IL-22), a member of the interleukin-10 cytokine family, has emerged as a vital mediator in maintaining the intestinal barrier [2]. IL-22 increases the expression of intestinal tight junctions to maintain the intestinal epithelial barrier and promotes the production of mucins and antimicrobial peptides to strengthen mucosal homeostasis [3,4]. Hence, IL-22 production is essential to preserve intestinal barrier integrity.

Innate lymphoid cells (ILC) are one type of immune cell that possesses the morphological characteristics of lymphocytes but lacks rearranged antigen receptors. As a member of ILCs, group 3 innate lymphoid cells (ILC3s), mainly located in intestinal lamina propria, are the primary secreted source of IL-22 [5]. Signal transducer and activator of transcription 3 (STAT3) is a transcription factor that is involved in the regulation of proliferation, survival, and apoptosis in many different types of cells. STAT3 signaling activation has been found to be a critical regulator for the production of IL-22 in ILC3s [6]. ILC3s have been found to produce IL-22 which in turn promotes the maintenance of intestinal barrier integrity and immunity homeostasis [7]. Signals from other resident cells and dietary factors can effectively promote ILC3 activation. Plant flavonoids are considered to be a type of natural aryl hydrocarbon receptor (AHR) ligand, and ILC3s could receive signals from dietary elements to modulate cell activation and cytokine secretion through AHR signaling [8]. Dihydromyricetin (DHM), a natural flavonoid mainly extracted from *Ampelopsis grossedentata*, has been found to exert therapeutic effects on metabolic diseases [9]. Previous studies indicate that DHM could alleviate intestinal inflammation and improve intestinal barrier integrity through remolding dysregulated gut microbiota [10]. However, the mechanism by which DHM ameliorates intestinal barrier dysfunction remains elusive. Sirtuin 3 (Sirt3) has been found to be a functional target of DHM to ameliorate HFD-induced nonalcoholic fatty liver disease (NAFLD) [11]. Sirt3 is an NAD-dependent deacetylase located mainly in mitochondria, and positively regulates fatty acid oxidation to exert protective effects on mitochondria [12,13]. The increased expression of genes related to fatty acid oxidation and mitochondrial respiratory function relative genes has been found to be associated with ILC3 activation [14]. We investigated whether DHM could promote IL-22 expression through STAT3 phosphorylation via SIRT3 signaling in ILC3s.

In this study, we showed that HFD feeding decreased the IL-22 expression to destroy the integrity of the intestinal epithelial barrier, and DHM repaired HFD-induced intestinal epithelial barrier destruction by inducing the IL-22 production in ILC3s. Within this regulation, DHM promoted IL-22 expression via STAT3 activation through SIRT3 signaling.

## 2. Materials and Methods

### 2.1. Animals and Treatment

Male wild type (WT) C57BL/6 6-week-old mice, obtained from Shanghai Model Organisms Center (Shanghai, China), were randomly divided into 3 groups, as follows: (1) Normal chow diet (NCD) group (n = 10): mice were fed a diet containing 10% of the available energy as fat; (2) High-fat diet (HFD) group (n = 10): mice were fed a diet containing 45% of available energy as fat; (3) HFD + DHM diet group (n = 10): mice were fed a diet containing 45% of available energy as fat and mixed with 0.6% DHM. All diets were provided by JIANGSU XIETONG PHARMACEUTICAL BIO-ENGINEERING CO., LTD. (Jiangsu, China). For IL-22 intervention, mice from the HFD and HFD + DHM groups were administered (i.p.) with 100 ng/g recombinant IL-22 (R&D systems, CA, USA) or phosphate-buffered saline (PBS) as a vehicle control in the final 4 weeks, twice a week as previously reported, respectively [15]. Animal experiments were carried out according to the animal care committee at the Third Military Medical University (Chongqing, China).

### 2.2. FITC-Dextran Permeability Assays

The FITC-dextran assay was performed after 12 weeks of feeding before the sacrifice of mice. The 4 kDa-FITC-dextran (Santa Clara, CA, USA) at a dosage of 600 mg/kg/B.W. was given to mice after fasting for 6 h. After 4 h of induction, a blood sample was collected from the caudal vein and centrifuged at 6000× *g* rpm for 10 min. The blood serum mixed with the same volume of PBS was detected via a SpectraMax M2 microplate reader (Molecular Devices, CA, USA). The permeability was calculated according to the fluorescence intensity based on a previous study [16].

### 2.3. Intestinal Lamina Propria Immune Cells Isolation

The small intestine collected from mice was washed with precooled PBS to remove feces and then cut into segments. Immune cells on lamina propria obtained from small intestinal tissues were resuspended in PBS for flow cytometry, according to our previous study [17].

### 2.4. Cell Culture and Treatments

MNK3 cells were cultured in a cell medium composed of DMEM, 10% FBS, and penicillin/streptomycin. For the construction of the SIRT3 knockdown cell line, MNK3 cells were co-cultured with SIRT3 knockdown lentivirus (LV-shSIRT3) or blank vector (LV-shNC) (Shanghai Genechem Co., Ltd, Shanghai, China.) for 12 h, and then the culture medium was replaced. The lentivirus-infected cells were screened in a culture medium containing puromycin for purification. To detect the effect of DHM and palmitic acid (PA) on MNK3 cells, cells were pretreated with DHM for 4 h followed by PA (0.2 mM) for another 12 h. To detect the involvement of AMPK, SIRT3, or STAT3 in DHM-induced benefits on MNK3 cells, cells were pretreated with inhibitors of SIRT3 (3-TYP, 50 μM; MCE, Newark, USA), AMPK (Dorsomorphin, 10 μM; MCE) or STAT3 (Static, 5 μM; Selleck Chemicals, TX, USA) for 2 h prior to the treatment of DHM and PA.

### 2.5. Flow Cytometry

Cells resuspended in PBS were stained with a series of mouse antibodies to detect ILC3s as follows: CD45 APC/Cyanine7 (30-F11) (Cat#: 103116, Biolegend, CA, USA), CD90.2 FITC (53–2.1) (Cat#: 103116, Biolegend), Lineage Cocktail Pacific Blue™ (17A2/RB6-8C5/RA3-6B2/Ter-119/M1/70) (Cat#: 133310, Biolegend), ROR gamma (t) PE (B2D) (Cat#: 12-6981-82e, Bioscience, Bristol, England), ROR gamma (t) APC (B2D) (Cat#: 17-6988-82, Bioscience), IL-22 PE (1H8PWSR) (Cat#: 12-7221-82, Bioscience), and L-22 PerCP-eFluor 710 (1H8PWSR) (Cat#: 46-7221-82, Bioscience). To measure transcription factor expression, cells were fixed and permeabilized using the Foxp3/Transcription Factor Staining Buffer Set (Invitrogen, CA, USA), according to the manufacturer’s instructions. For intracellular cytokine expression, cells were stimulated with 50 ng/mL PMA (Sigma), 500 ng/mL ionomycin (Sigma, MO, USA), 20 ng/mL IL-1β, 20 ng/mL IL-23 (Pepro tech, NJ, USA), and GolgiPlug (BD) for 4 h. Then, the cells were fixed and permeabilized using a Cytofix/Cytoperm™ Fixation/Permeabilization Solution Kit (BD, Newark, USA), according to the manufacturer’s instructions. After staining, the cells were resuspended with PBS for flow cytometry analysis via LSR Fortessa (BD) or Verse (BD).

### 2.6. Histological Assessment

The intestinal and colon tissues were harvested and fixed in 4% paraformaldehyde immediately after the mice were sacrificed, embedded into paraffin, and then cut into 3–10 µm slices. Subsequently, paraffin sections were deparaffinized and stained with hematoxylin and eosin (H&E) staining. The stained sections were examined and imaged using a microscope (Leica, Germany).

### 2.7. Immunoblotting

The intestinal tissues collected from mice were lysed in tissue lysis buffer (Thermo, MA, USA) supplemented with a complete protease inhibitor cocktail (Roche, NJ, USA). The protein concentration of the extracts was determined using a bicinchoninic cid assay (BCA). The same amount of protein from different groups was subjected to electrophoretic analysis and transferred onto polyvinylidene fluoride (PVDF) membranes. The membranes were incubated with primary antibody overnight at 4 °C, then treated with the horseradish peroxidase (HRP)-labeled secondary antibody at room temperature for 60 min. The expression of β-actin was applied as a control in the quantitative analysis. Results were visualized using an immobilon-enhanced chemiluminescence (ECL) Ultra Western HRP Substrate (Millipore, MA, USA). The expression of the protein was determined with Quantity One Image software v1.8.0 (Bio-Rad). The following antibodies were used: SIRT3 (#2627, 1:1000, Cell Signaling Technology, MA, USA), SIRT3 (ab246522, 1:1000, Abcam, Cambridge, England), Zo-1 (ab276131, 1:1000, Abcam), and Claudin (ab211737, 1:1000, Abcam). HRP-conjugated anti-mouse or anti-rabbit secondary antibodies were obtained from Invitrogen.

### 2.8. Quantitative Real-Time PCR

The total RNA was isolated from tissues or cells using TRIzol (Invitrogen). A NanoDrop spectrophotometer was used to detect RNA concentration. Reverse transcription for cDNA synthesis was conducted with a PrimeScript RT Master Mix (Takara). Quantitative real-time PCR was performed using an SYBR RT-PCR kit (Takara), and the results were analyzed with a qTOWER 2.2 system (Anakytik Jena, Germany). The expressions of target genes were normalized to those of 18s rRNA or β-actin. The primer sequences for the target genes are listed in Table 1.

### 2.9. Statistical Analysis

The data were analyzed using SPSS 19.0 software and are presented as the mean ± SEM. Statistical differences among groups were determined with one-way ANOVA followed by the Tukey–Kramer post hoc test. *p* values less than 0.05 were considered statistically significant.

## 3. Results

### 3.1. HFD-Induced Intestinal Epithelial Barrier Destruction Was Associated with the Down-Expression of IL-22

**Figure 1:**
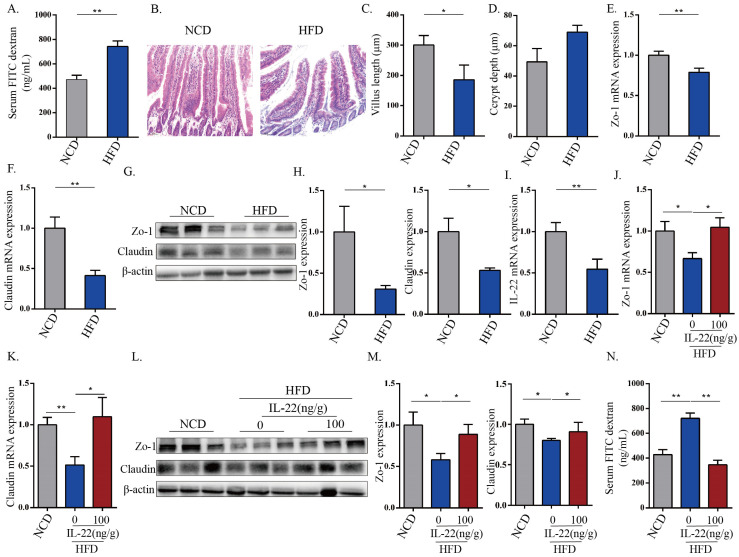
HFD-induced intestinal epithelial barrier destruction was associated with the down-expression of IL-22. (**A**) Serum FITC–dextran (4 kD) intensities of the indicated groups. (**B**) Hematoxylin and eosin (H&E) staining of the small intestinal tissues. Scale bars = 50 μm. (**C**) Villus length. (**D**) Crypt depths of small intestinal tissues in the indicated group were calculated according to HE images. The mRNA expressions of Zo-1 (**E**) and claudin (**F**) in small intestine tissues. (**G**,**H**) The protein expression levels of ZO-1 and claudin in small intestine tissues of the indicated mice were calculated via Western blotting (3 samples/group). β-actin was used as a loading control. (**I**) Evaluation of the relative mRNA expression of IL-22 in small intestine tissues of the indicated mice were calculated using qPCR. (**J**–**M**) The mRNA and protein expression levels of ZO-1 and claudin in small intestine tissues were detected after treatment with IL-22. (**N**) The intestinal barrier permeability was calculated via serum FITC–dextran (4 kD) intensities after treatment with IL-22. Mean ± SEM are plotted. * *p* < 0.05, ** *p* < 0.01, as determined by one-way ANOVA followed by the Tukey–Kramer post hoc test.

To investigate the effect of HFD on the intestinal epithelial barrier, mice were fed an NCD or HFD for 12 weeks. Leakage of the intestinal barrier is a critical step in pathophysiological conditions; we first detected the intestinal permeability of mice in the indicated groups. Results showed that intestinal permeability was significantly elevated after HFD feeding (Figure 1A). The small intestinal morphology was analyzed via H&E staining (Figure 1B), which showed that HFD feeding resulted in a significantly reduced length of intestinal villi (Figure 1C). The length of crypt depth was increased after HFD feeding, whilst no significant changes were observed in the two groups (Figure 1D). Since tight junction proteins (TJ) connect intestinal epithelial cells to maintain barrier integrity [18], we next evaluated the impact of HFD feeding on TJ expression. As shown in Figure 1E–H, the mRNA and protein expressions of Zo-1 and claudin decreased after HFD feeding. Since IL-22 is an important cytokine involved with the maintenance of intestinal epithelial cells and gut barrier integrity [15], we hypothesized that HFD feeding may decrease IL-22 expression in the small intestine, and that HFD-fed mice treated with IL-22 would show improved intestinal barrier function. As shown in Figure 1I, IL-22 mRNA expression in the small intestine significantly decreased in HFD-fed mice. Furthermore, IL-22 treatment induced a significant increase in Zo-1 and claudin protein expressions (Figure 1J–M), which coincided with an improvement in the intestinal permeability induced by IL-22 treatment (Figure 1N). Overall, these results indicate that an HFD induces obvious intestinal epithelial barrier destruction, which is associated with a decreased expression of IL-22; meanwhile, IL-22 treatment, in turn, obviously ameliorates HFD-induced epithelial barrier destruction.

### 3.2. DHM Protected against HFD-Induced Intestinal Barrier Destruction That Was Associated with Increased IL-22 and ILC3 Frequency

**Figure 2:**
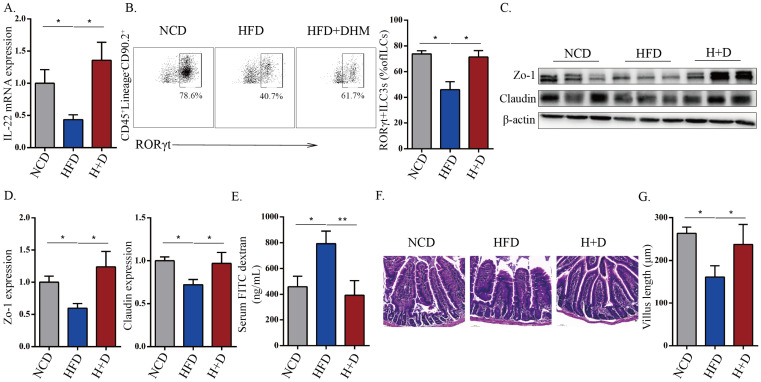
DHM protected against HFD-induced intestinal barrier destruction, which was associated with an increase in IL-22, and increased ILC3 frequency. (**A**) The relative expression of IL-22 in small intestine tissues of the indicated mice was calculated via qRT-PCR. (**B**) Calculation of the frequency of RORγt+ ILC3 cells (CD45 + lineage-CD90.2 + RORγt+) derived from the small intestine in the indicated mice. The numbers in the flow plots represent a percentage of ILC3s in each gate. (**C**,**D**) The mRNA and protein expression levels of ZO-1 and claudin in small intestine tissues of the indicated mice were calculated via Western blotting (3 samples/group). β-actin was used as a loading control. (**E**) Serum FITC–dextran (4 kD) intensities of indicated mice. (**F**) Hematoxylin and eosin (H&E) of the small intestinal tissues. Scale bars = 50 μm. (**G**) Villus length of small intestinal tissues in the indicated mice was calculated according to (**F**). * *p* < 0.05, ** *p* < 0.01, as determined by one-way ANOVA followed by the Tukey–Kramer post hoc test.

As we showed, IL-22 treatment increased the expression of TJ proteins to ameliorate HFD-induced intestinal epithelial barrier destruction. We next explored whether IL-22 was involved in the protection of intestinal barrier integrity with DHM treatment. The IL-22 mRNA expression in small intestinal tissue was detected after mice were fed with NCD, HFD, and HFD plus DHM for 12 weeks. Mice fed with HFD plus DHM showed a significantly higher mRNA expression of IL-22 than that in the HFD group (Figure 2A). Moreover, ILC3s mainly resided in the intestinal lamina propria, which are the main source of IL-22, contributing to the protection of intestinal barrier integrity [19,20]. As IL-22 mRNA expression in the intestinal tissue increased in HFD plus DHM-fed mice (Figure 2A), the effect of DHM on the percentage of ILC3s in the intestine was assessed. Briefly, the lymphocytes in the small intestinal lamina propria were isolated from mice, and ILC3s were analyzed with flow cytometry. The percentage of CD45 + Lineage-CD90.2 + RORγt + ILC3s significantly decreased in the HFD group compared with that in the control (Figure 2B), in which the inhibition was obvious after DHM treatment (Figure 2B). Furthermore, DHM administration noticeably inhibited HFD-induced decreases in mRNA and protein expressions of Zo-1 and claudin (Figure 2C,D), increased FITC-dextran leakage induced by HFD (Figure 2E), as well as reduced the length of intestinal villi induced by HFD (Figure 2F), suggesting there was a protective effect of DHM against HFD-induced intestinal barrier destruction. Taken together, these results indicate that DHM could protect against intestinal barrier destruction induced by HFD, which is associated with an increased expression of IL-22 and ILC3 frequency in the intestine.

### 3.3. DHM Promoted IL-22 Expression in MNK3 Cells through SIRT3 via AMPK/PGC1α Signaling

**Figure 3:**
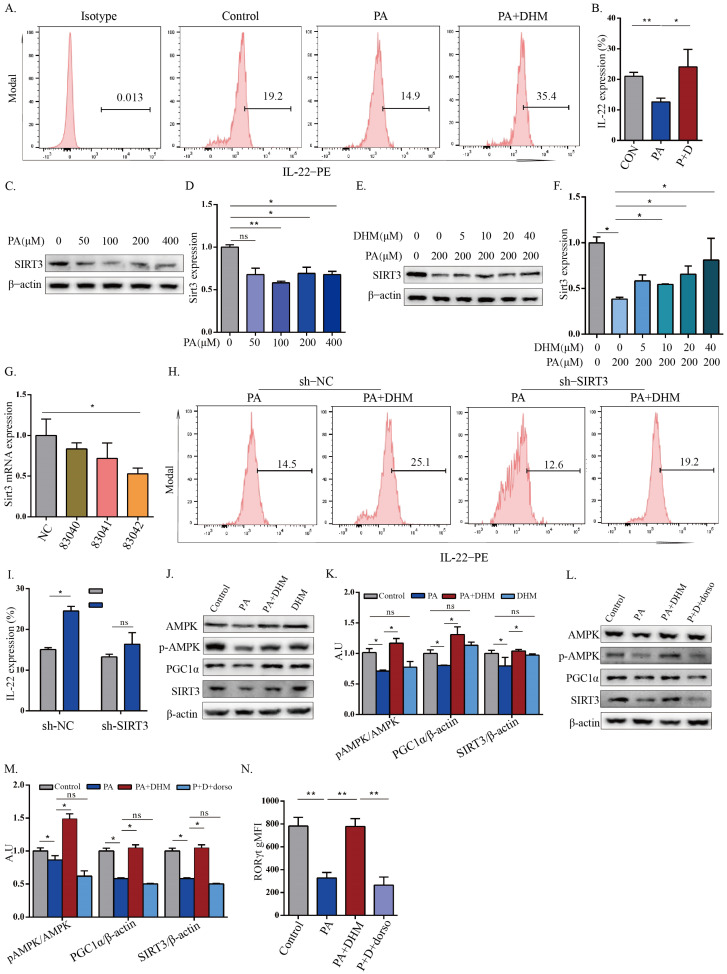
DHM promoted IL-22 expression in MNK3 cells through SIRT3 via AMPK/PGC1α signaling. (**A**,**B**) IL-22 expression in the indicated groups was detected by flow cytometry. (**C**,**D**) The protein expression levels of SIRT3 in MNK3 cells after treatment with different concentrations of PA were detected with Western blotting. (**E**,**F**) The protein expression levels of SIRT3 in MNK3 cells treated with PA in the presence or absence of DHM were detected via Western blotting. (**G**) The mRNA expression of SIRT3 in MNK3 cells with SIRT3 lentivirus infection. (**H**,**I**) IL-22 expression in the indicated groups was detected using flow cytometry. (**J**,**K**) Representative immunoblots of AMPK, p-AMPK, PGC1α, and SIRT3 in the indicated groups, and quantitative graphs show the ratios of p-AMPK/AMPK, PGC1α/β-actin, and SIRT3/β-actin. (**L**,**M**) Representative immunoblots of AMPK, p-AMPK, PGC1α, and SIRT3 after pretreatment with AMPK inhibitor, and quantitative graphs show the ratios of p-AMPK/AMPK, PGC1α/β-actin, and SIRT3/β-actin. (**N**) The RORγt expression levels were detected using flow cytometry. * *p* < 0.05; ** *p* < 0.01; ns, non-significance as determined by one-way ANOVA followed by the Tukey–Kramer post hoc test.

Next, we explored the underlying mechanisms by which DHM promoted increased IL-22 expression in ILC3s. The MNK3 cell line, which expresses the same transcription factors and cytokines as RORγt and IL-22 as ILC3s, was applied in this study. The MNK3 cells were induced with PA with or without DHM, and the potential involvement of SIRT3 in the modulation of IL-22 expression was investigated. As shown in Figure 3A,B, IL-1β/IL-23-stimulated MNK3 cells showed lower expression levels of RORγt and IL-22 under PA treatment, which was significantly inhibited by DHM treatment. The expression of SIRT3 dramatically decreased in PA-treated MNK3 cells (Figure 3C,D), which was obviously recovered by DHM treatment (Figure 3E,F) in a dose-dependent manner, implying that the DHM-induced increase in IL-22 secretion may be associated with an upregulation of SIRT3 expression in MNK3 cells. Then, an SIRT3 knockdown MNK3 cell line was developed to assess the involvement of SIRT3 in IL-22 expression. As shown in Figure 3G, the mRNA levels of SIRT3 noticeably decreased in SIRT3 knockdown MNK3 cells with different SIRT3 knockdown lentivirus, and the 83,042 lentiviruses were selected according to their optimal effect. DHM effectively recovered the reduced IL-22 expression caused by PA treatment (Figure 3H,I), while these effects were obstructed after SIRT3 inhibition, suggesting that SIRT3 was substantially involved in the regulation of IL-22 expression by DHM treatment. A previous study demonstrated that AMPK and PGC1α could modulate SIRT3 expression [21]. Then, we further detected whether DHM regulated SIRT3 expression through modulation of AMPK or PGC1α in MNK3 cells. As expected, DHM administration significantly ameliorated the decreased phosphorylation of AMPK and expression of PGC1α caused by PA (Figure 3J,K). However, when MNK3 cells were pretreated with an AMPK inhibitor, dorsomorphin, the recovery effects of DHM on PA-triggered attenuation on SIRT3, AMPK phosphorylation, and PGC1α expression were abolished (Figure 3L,M). The increased MNK3 activation by DHM was also abrogated after AMPK blockage, as measured by RORγt expression via flow cytometry (Figure 3N). Collectively, our findings indicate that DHM promotes IL-22 expression in MNK3 cells through the AMPK/PGC1α/SIRT3 signaling pathway.

### 3.4. DHM Promoted IL-22 Expression in MNK3 Cells via STAT3 Phosphorylation

**Figure 4:**
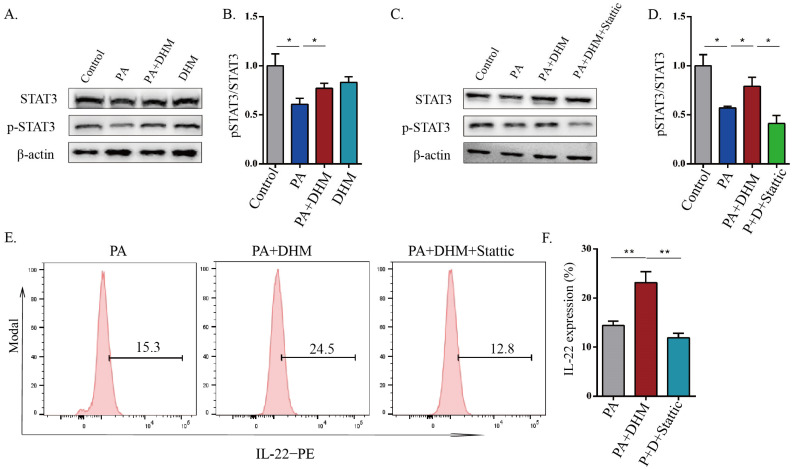
DHM promoted IL-22 expression in MNK3 cells via STAT3 phosphorylation. (**A**,**B**) Representative immunoblots of STAT3 and p-STAT3 in the indicated groups, and quantitative graphs show the ratios of p-STAT3/STAT3. (**C**,**D**) The protein expressions of STAT3 and p-STAT3 after pretreatment with STAT3 inhibitor Static. (**E**,**F**) IL-22 expression in the indicated groups was detected with flow cytometry. * *p* < 0.05, ** *p* < 0.01, as determined by one-way ANOVA followed by the Tukey–Kramer post hoc test.

STAT3, a member of the STAT (signal transducers and activators of transcription) family whose activation shapes IL-22 expression [22,23], was used to gain insight into the molecular mechanism under IL-22 expression by DHM treatment. As shown in Figure 4A,B, STAT3 phosphorylation significantly decreased in PA-treated MNK3 cells, and was effectively inhibited by DHM treatment. To further investigate whether DHM enhanced IL-22 expression via STAT3 phosphorylation, a STAT3 inhibitor static (5 μM) was employed. After being pretreated with STAT3 inhibitor, the effects of DHM on STAT3 phosphorylation and IL-22 expression in MNK3 cells were significantly attenuated (Figure 4C–F). Taken together, the results indicate that the promotional effects of DHM on IL-22 expression may be mediated through the increased phosphorylation of STAT3.

### 3.5. DHM Stimulated STAT3 Activation through SIRT3 in MNK3 Cells via ERK-CREB Signaling

**Figure 5:**
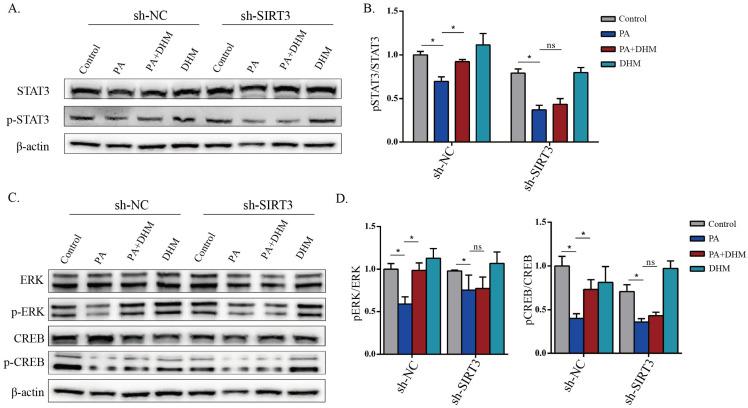
DHM stimulated STAT3 activation through SIRT3 in MNK3 cells via ERK-CREB signaling. (**A**,**B**) Representative immunoblots of STAT3 and p-STAT3 in the indicated groups, and quantitative graphs show the ratios of p-STAT3/STAT3. (**C**) The protein expression levels of ERK, p-ERK, CREB, and p-CREB in the indicated groups were detected using Western blotting. (**D**) Quantitative graphs show the ratios of p-ERK/ERK and p-CREB/CREB. * *p* < 0.05; ns, non-significance as determined by one-way ANOVA followed by the Tukey–Kramer post hoc test.

The above findings demonstrated that SIRT3 and STAT3 may play crucial roles in DHM-mediated stimulative effects on IL-22 expression in MNK3 cells; the potential mechanisms require further clarification. We hypothesized that DHM could promote IL-22 secretion of MNK3 cells via SIRT3-mediated STAT3 activation. To verify this hypothesis, an SIRT3 knockdown MNK3 cell line was applied. DHM effectively inhibited the reduced STAT3 phosphorylation caused by PA treatment, while these effects were obstructed by SIRT3 knockdown (Figure 5A,B). To clarify the molecular mechanism by which SIRT3 regulates STAT3, we concentrated on the ERK/CREB signaling pathway, since it was demonstrated to be associated with STAT3 and ILC3-derived IL-22 expression [23,24]. Our results illustrated that ERK and CREB phosphorylation decreased by PA treatment, which indicated that PA inactivated the ERK-CREB pathway, and ERK-CREB signaling pathway inactivation could be inhibited by DHM administration (Figure 5C,D). Interestingly, SIRT3 knockdown noticeably reversed the increased levels of phosphorylated ERK and CREB by DHM treatment (Figure 5C,D). In conclusion, DHM stimulates STAT3 activation in MNK3 cells through SIRT3, which may be mediated partially through the ERK-CREB signaling pathway.

## 4. Discussion

The intestinal barrier protects the host from the harmful effects of toxins, food antigens, and gut microbiota in the gastrointestinal tract. However, intestinal barrier integrity can be affected by multiple factors, including irrational lifestyle habits and diets [25]. Research indicates that HFD could negatively modulate intestinal barrier integrity by stimulating barrier-disrupting signal transduction to promote chronic metabolic disease [16]. The destruction and increasing intestinal permeability of this barrier are also related to intestinal pathologies, such as inflammatory bowel disease, irritable bowel syndrome, and celiac disease [26]. Moreover, intestinal inflammation has been demonstrated to be a potential stimulating factor associated with diet and/or obesity with related metabolic disorders [27]. However, there is no effective pharmacological treatment to restore intestinal barrier function [28]. Dietary strategies have been strongly recommended as a promising preventive treatment method. Currently, accumulating evidence suggests that flavonoids exert a protective role on intestinal barrier integrity by shaping microbiota populations, protecting the intestinal epithelium, and modulating the intestinal immune system [29,30,31]. Thus, flavonoid-rich diets are beneficial for the protection of intestinal barrier integrity. Previous studies have demonstrated that DHM, as a major flavonoid in *Ampelopsis grossedentata*, has been found to alleviate colitis by positively modulating intestinal barrier integrity [10,32]. In the present study, we further proved that DHM could effectively ameliorate HFD-induced TJ down-expression and increase gut permeability. However, the underlying mechanisms of the protective effects on the intestinal barrier elicited by DHM have not been fully clarified. Thus, our study aimed to analyze the molecular mechanism of DHM-derived protective effects on the intestinal barrier.

IL-22, a central regulator of epithelial homeostasis, is involved in the modulation of epithelial barrier function, such as stimulation of intestinal epithelial cell growth and turnover, induction of TJ, and mucus production [3,6,33,34,35]. Previous studies indicated that HFD induced intestinal inflammation and impaired gut barrier function, and that these adverse effects could be reversed by recombinant IL-22 [15]. The current findings confirmed these conclusions, and furthermore demonstrated that DHM could effectively alleviate increased intestinal barrier permeability, reduce expression of TJ proteins and destroy intestinal epithelial structure induced by HFD, and that these beneficial effects were associated with IL-22 expression in ILC3s (Figure 1 and Figure 2). IL-22 is primarily produced by ILC3s that settle in the intestinal mucosa. In addition, it can also be secreted by Th17 cells when needed [35,36]. ILC3s are mainly involved in mediating intestinal immune homeostasis by secreting a series of cytokines and surface receptors, one of which is to maintain intestinal barrier integrity under the balanced tuning between the enhancement or inhibition of the immune response against microbiota [37]. ILC3s are generated from a lymphoid progenitor in bone marrow, and mature ILC3s develop in the intestinal lamina propria under the regulation of specific differentiation factors, such as the microbiota and food [38,39]. Stimulating factors from microbiota metabolism products and diets could combine with the retinoic acid receptor (RAR), free fatty acid receptor (FFAR), and hydrocarbon receptor (AhR) to trigger ILC3s [8,40,41,42]. Research showed that the expressions of CYP1A1 and CYP1A2 are the activation markers of AHR signaling, which can be increased by DHM in the small intestine [43]. This indicates that DHM may be a potential regulator of ILC3s. In this study, we confirmed that DHM could effectively promote IL-22 expression in ILC3s (Figure 2 and Figure 3).

**Figure 6:**
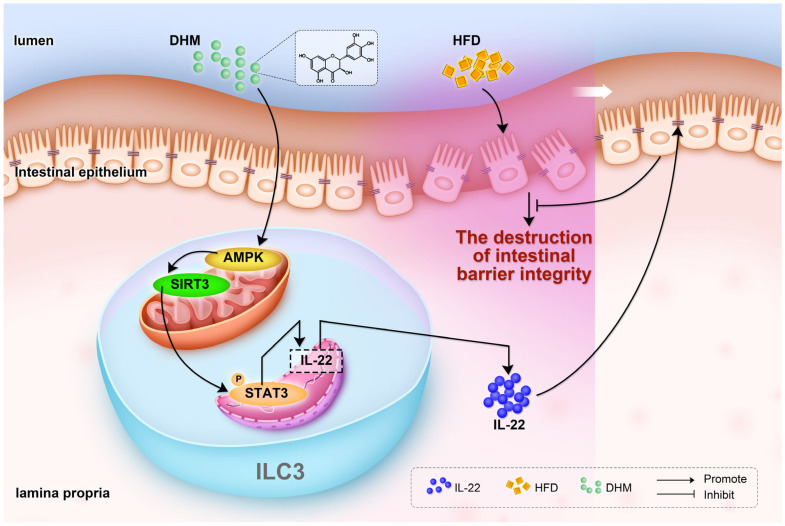
Model of DHM-mediated protective effects against HFD-induced intestinal barrier dysfunction.

After having confirmed that the DHM exerted protective effects on gut barrier integrity via enhancing IL-22 secretion in ILC3s in the small intestine, we attempted to explore the involved mechanism. Our previous study confirmed that SIRT3 is a functional target of DHM for its prevention and treatment of metabolic diseases [11]. SIRT3 knockout mice exhibited an exacerbated metabolic syndrome, disturbed gut microbial balance, and impaired intestinal permeability [44,45]. SIRT3, an NAD+-dependent deacetylase resident in the mitochondria, regulates metabolic homeostasis, glucose and fatty acid metabolism, and oxidative stress through regulating protein lysine deacetylation [46]. In addition, mitochondrial oxidation of lipids has been found to activate ILC3s, and activated ILC3s possess an increase in the expression of genes associated with lipogenesis, glycolytic mediators, and mitochondrial respiratory function [14]. The present study indicated that DHM could promote IL-22 production in ILC3s by enhancing SIRT3 expression (Figure 3 and Figure 6). In order to further illustrate the underlying mechanism associated with the effects of DHM on SIRT3 expression in ILC3s, we also assessed whether DHM regulated SIRT3 expression via AMPK. AMPK, a pivotal modulator in mitochondrial metabolism, plays a critical role in regulating T-cell metabolism [47]. Moreover, AMPK has been found to modulate SIRT3 signaling for T-cell memory development [48,49]. In this study, we also found that AMPK inhibition significantly abolished the effects of DHM on SIRT3 and IL-22 expression in ILC3s (Figure 3). Our results indicate that DHM can promote IL-22 expression of ILC3s via AMPK/SIRT3 signaling.

It has been proven that STAT3 activation induces the induction of RORγt and IL-22 in T cells [35]. Consistent with this study, our current findings demonstrated that the phosphorylation of STAT3 in ILC3s was upregulated by DHM intervention, and the function of DHM on IL-22 expression was weakened after STAT3 inhibition (Figure 4). Previous studies showed that ERK signaling could promote STAT3 phosphorylation to enhance IL-22 production in ILC3s [23]. Moreover, HFD downregulates SIRT3 to inactivate the ERK-CREB pathway to trigger metabolic diseases [50]. Our data demonstrate that, in addition to STAT3 activation and IL-22 expression induced by DHM in ILC3s, DHM enhanced SIRT3 expression to activate ERK/CREB signals, leading to STAT3 activation and IL-22 secretion (Figure 5).

## 5. Conclusions

In summary, our results indicate that DHM alleviated HFD-induced intestinal barrier destruction via the promotion of IL-22 expression in ILC3s. This beneficial effect was involved with the enhanced expression of SIRT3 expression and ERK/CREB pathway activation induced by SIRT3, thereby leading to the phosphorylation of STAT3 and IL-22 secretion. Since the intestinal barrier is associated with multiple types of metabolic dysfunction, our results demonstrate a potential mechanism under DHM-mediated protective effects on metabolic disease that provides supporting theories for its usage in dietary strategy.

## Figures and Tables

**Table 1:** Primers used in this study.

	Forward Primers (5′→3′)	Reverse Primers (5′→3′)
zo-1	AGGACACCAAAGCATGTGAG	GGCATTCCTGCTGGTTACA
occludin	TTGAAAGTCCACCTCCTTACAGA	CCGGATAAAAAGAGTACGCTGG
sirt3	CGCTAAACTTCTCCCGGGTT	ACACTAGTCCTCGCCAAACG
Il22	CATGCAGGAGGTGGTACCTT	CAGACGCAAGCATTTCTCAG
18sRNA	ACGGACCAGAGCGAAAGCAT	TGTCAATCCTGTCCGTGTCC

## Data Availability

Not applicable.
